# Chemical Composition Analysis Using HPLC-UV/GC-MS and Inhibitory Activity of Different *Nigella sativa* Fractions on Pancreatic *α*-Amylase and Intestinal Glucose Absorption

**DOI:** 10.1155/2021/9979419

**Published:** 2021-06-26

**Authors:** Mohammed Dalli, Nour Elhouda Daoudi, Salah-eddine Azizi, Hind Benouda, Mohamed Bnouham, Nadia Gseyra

**Affiliations:** ^1^Laboratory of Bioresources, Biotechnology, Ethnopharmacology and Health, Unit of Physiology and Ethnopharmacology, University Mohamed Premier, Faculty of Sciences, Oujda, Morocco; ^2^Laboratory of Organic Chemistry, Macromolecules and Natural Products, University Mohamed Premier, Faculty of Sciences, Oujda, Morocco

## Abstract

*Nigella sativa* (NS) is a well-known plant for its various benefits and multiuse in traditional medicine. This study is aimed at investigating the chemical composition of the different *NS* fractions by using GC-MS for the esterified fatty acids or HPLC-UV for organic fraction and at evaluating the inhibitory effect on pancreatic *α*-amylase (*in vitro*, *in vivo*) and intestinal glucose absorption. Among all the investigated fractions, it was shown that they are rich with different molecules of great interest. The n-hexane fraction was characterized by the presence of linoleic acid (44.65%), palmitic acid (16.32%), stearic acid (14.60%), and thymoquinone (8.7%), while among the identified peaks in EtOH fraction we found catechin (89.03 mg/100 g DW), rutin (6.46 mg/100 g DW), and kaempferol (0.032 mg/100 g DW). The MeOH fraction was distinguished with the presence of gallic acid (19.91 mg/100 g DW), catechin (13.79 mg/100 g DW), and rutin (21.07 mg/100 g DW). Finally, the aqueous fraction was marked by the existence of different molecules; among them, we mention salicylic acid (32.26 mg/100 g DW), rutin (21.46 mg/100 g DW), and vanillic acid (3.81 mg/100 g DW). Concerning the inhibitory effect on pancreatic *α*-amylase, it was found that in the *in vitro* study, the best IC_50_ registered were those of EtOH (0.25 mg/ml), MeOH (0.10 mg/ml), aqueous (0.031 mg/ml), and n-hexane fraction (0.76 mg/ml), while in the *in vivo* study an important inhibition of *α*-amylase in normal and diabetic rats was observed. Finally, the percentage of intestinal glucose absorption was evaluated for all tested extracts and it was ranging from 24.82 to 60.12%. The results of the present study showed that the *NS* seed fractions exert an interesting inhibitory effect of *α*-amylase and intestinal glucose absorption activity which could be associated with the existent bioactive compounds. Indeed, these compounds can be used as antidiabetic agents because of their nontoxic effect and high efficacy.

## 1. Introduction

Diabetes mellitus is a metabolic disorder considered abnormal glucose homeostasis that is characterized by an increase in blood sugar. This serious disease affects a large number of populations in the world and is characterized by a continuous increase with aging because of the increasing prevalence of obesity and sedentary lifestyle [1]. The prevalence of diabetes will be increasing to 50% between 2000 and 2030 [2]. Two types of diabetes are mainly known: the type 1 which is related to the deficiency of insulin and type 2 that has a direct relation with insulin resistance [3].

The gastrointestinal tract is very rich in different digestive enzymes such as *α*-amylase that is endowed with a great capacity to split the *α*-1,4 of the glycosidic linkage in starch which induces the formation of maltose and glucose [4]. Generally, there are two types of *α*-amylase enzyme: the salivary and the pancreatic one which continues the starch digestion on the small intestine. Its inhibition is considered an important target in the regulation of postprandial blood glucose increase in diabetes [5]. The mechanism behind the intestinal glucose absorption and transport from the luminal side to the bloodstream is considered an important path for the prevention and treatment of insulin-resistant diabetic patients [6].

*Nigella sativa* (*NS*) also known as black cumin is a plant belonging to the Ranunculaceae family, a well-distributed plant in North Africa, Middle East, Europe, and Asia [7]. The black cumin possesses a large spectrum of effects such as immunomodulatory [8], antitumoral [9, 10], and antifungal [11], plus its antihypertensive activity [12]. The plant has been considered as a rich source for different secondary metabolites such as polyphenols, flavonoids, tannins, saponins, and alkaloids [13]. Recently, it was proved that *NS* is endowed with an antioxidant [14] and antibacterial activity [15, 16]. The black cumin has a great capacity to lower glucose by increasing the insulin level; also, it was found to decrease glycated hemoglobin [17], which made this plant among the promising alternatives that can be used in the prevention and treatment as antidiabetic agents [18].

The present study is a continuity of our previous study, and it is aimed at elucidating the action mechanism behind the antidiabetic effect of *NS* fractions by studying their inhibitory actions on pancreatic *α*-amylase (*in vitro*/*in vivo*) and the *in situ* intestinal glucose absorption. The HPLC/UV and the GC/MS were used to determine the different bioactive compounds present and could contribute into the studied pharmacological effect.

## 2. Material and Methods

### 2.1. Plant Material

*Nigella sativa* seeds were purchased from the local market. For the accuracy of the work, the botanical identification of the plant material was assessed at the Faculty of Sciences Oujda, and a specimen was deposited at the faculty herbarium under the voucher number HUMPOM471.

### 2.2. Preparation of *Nigella sativa*

The *NS* seeds were cleaned from dust and residues and then left dried in a dark room for about a week. The dried seeds were then turned into fine powder before extraction. After that, 100 g was taken and extracted using different solvents of increasing polarities starting from hexane to water using the Soxhlet apparatus. After each extraction, the obtained fractions were dried using a rotary evaporator. The obtained fractions were put at 4°C for further use.

### 2.3. Qualitative and Semiquantitative GC-MS Analysis

The preparation of the methyl esters was realized according to the protocol NF T60-233 [19]. The esterified n-hexane fraction was analyzed using a gas chromatograph (Shimadzu GC-2010) equipped with a fused-silica capillary column (5% phenyl methyl siloxane, 30 m × 0.25 mm, 0.25 *μ*m film thickness) coupled with a mass spectrometer detector (GC-MS-QP2010). The helium as a carrier gas was adjusted to a constant pressure of 100 kPa. The oven temperature was set initially at 50°C (maintained for 1 minute) and followed by a gradient of 10°C/min up to 250°C (maintained for 1 minute). The temperatures of the injector, transfer line, and ion source were set at 250°C, 250°C, and 200°C, respectively. For the qualitative and semiqualitative analysis, solutions containing 1 *μ*l of the samples diluted in hexane (50 mg/g) were injected in split mode (split ratio = 50–80) and the GC-MS system was operated in scan mode. Mass spectra were recorded at 70 eV (electron impact ionization mode) with an *m*/*z* range of 40-350 a.m.u. (rate and solvent delays were 5 s/scan and 4.5 minutes, respectively). Identification of the fatty acid constituents was accomplished based on the comparison of their MS data with those stored on the National Institute of Standards and Technology (NIST147) computer library. LabSolutions (version 2.5) was used for data collection and processing.

### 2.4. Qualitative and Quantitative HPLC Analysis

For the determination of different HPLC profiles, the different fractions (aqueous, MeOH, and EtOH) were prepared at a concentration of 20 mg/ml. After that, a filtration through 20 *μ*m Millipore filters has been done. Then, 20 *μ*l of each sample was taken and injected into Alliance ew2695, C_18_ (250 × 4.0 mm, 5 *μ*m) reversed-phase column of a high-performance liquid chromatography system that is connected to a UV detector PDA Waters 2996 (210-400 nm). The HPLC analysis was performed using a linear gradient starting from 80% water in acetic acid to 100% of methanol for 20 min, followed by 100% for 25 min with a flow rate of 1 ml/min, UV detection at 254 nm. The peak areas and heights were analyzed by Empower (version 3), a software provided with HPLC. Concerning the HPLC profile, different chemicals were used such as gallic acid, vanillic acid, naringenin, rutin, catechin, kaempferol, vanillin, ferulic acid, and salicylic acid. The different analytic standards were prepared in DMSO (1 mg/ml); 10 *μ*l was injected into the system using the same protocol described above. The contents of the phenolic compounds were quantified from the calibration curve of each standard and are expressed in mg/100 g of dry weight. The analysis was carried out in triplicates. All calibration curves showed good linearity *r*^2^ > 0.99.

### 2.5. Animals

The *albino* mice and *Wistar* rats used in the experiment were taken from the animal facility at the Department of Biology, Faculty of Sciences Oujda, Morocco. The animals were kept in appropriate cages in a well-ventilated room with free access to food and water and with respect to the laboratory standard conditions (light/dark cycle of 12 h/12 h and a temperature of 24 ± 2°C). All animals were cared according to the international guidelines for the care and use of laboratory animals, published by the US National Institutes of Health [20].

### 2.6. In Vitro Inhibition of *α*-Amylase

The inhibition capacity of the different fractions obtained from the Soxhlet apparatus on *α*-amylase enzyme was performed according to Thalapaneni et al. [21]. The reaction mixture contains 200 *μ*l of the *NS* fractions or acarbose used as control at different concentrations (0.45, 0.9, and 1.82 mg/ml), 200 *μ*l of the phosphate buffer (pH = 6.9), and 200 *μ*l of the *α*-amylase enzyme solution. The reaction mixture was preincubated for 10 min at 37°C. After that, 200 *μ*l of starch 1% was added to each tube and the mixture was then incubated for 20 min at 37°C. To stop the reaction, DNSA color reagent (600 *μ*l) was added. Afterward, all tubes were put to be incubated at 100°C for 8 min and then were cooled in a cold-water bath. Finally, the different tubes of the tested fraction and control were diluted using 1 ml of distilled water. The absorbance of the mixture was measured at 540 nm.

The inhibition percentage was calculated using the following formula:

(1) $$\begin{array}{c} \text{ percentage of inhibition activity }\left(\%\right)=\frac{\left(\text{ODtest }540\text{nm}-\text{ODcontrol }540\text{nm}\right)}{\text{OD test }540\text{nm}}\times 100 \end{array}$$

### 2.7. Acute Toxicity Test

The guidelines for testing chemicals (2008) of the Organization for Economic Cooperation and Development have been strictly followed for testing the acute oral toxicity [22]. A batch of 30 mice was divided into 5 groups, with 6 mice each (3 males/3 females): the first group represents the control group, which receives the distilled water. The remaining 3 groups were treated with increasing doses 0.5 g/kg, 2 g/kg, and 5 g/kg of the different fractions; then, they were individually observed for the first 30 minutes and then regularly for the early 24 h and daily for 14 days of toxicity study.

### 2.8. Diabetes Induction

Diabetes was induced according to the procedure described by [23]. The animals were fasted for about 16 h with accessibility to water. After that, all animals were injected intraperitoneally with alloxan (140 mg/kg) dissolved in phosphate sodium-citrate buffer (pH = 3). One week after, the administration was verified using a glucose oxidase-peroxidase method. Glycemia was found to be higher than 1.5 g/l.

### 2.9. In Vivo Inhibition of *α*-Amylase Enzyme

This study was performed using normal and diabetic rats with a weight ranging from 200 to 300 g. 16 h before the experimentation, all animals were deprived of food with free access to water. The animals were divided into four groups (*n* = 6; ♂/♀ = 1); each group received per os a dose of 250 mg/kg from each fraction. The positive control group has received acarbose at a dose of 10 mg/ml using the same route of administration. The control group received distilled water (10 ml/kg). The animals were loaded orally with starch (2 g/kg) after 30 min of fraction administration. The glycemic level was estimated at different times (0, 30, 60, and 120 min) using a glucose oxidase-peroxidase method.

### 2.10. In Situ Intestinal Glucose Absorption

The following experiment was realized according to [24]. This technique is based on the use of the *Wistar* rat jejunum. Before the experiments, all animals were fasted for about 36 h with free access to water. At the day of the experiment, the animals were anesthetized using intramuscular injection of pentobarbital (50 mg/kg). 10 cm of the jejunum is then perfused with the appropriate perfusion solution+glucose in the control group while in the positive control the jejunum was perfused using phlorizin. Concerning the tested fractions, the jejunum was perfused of each fraction with a dose of 250 mg/kg. The perfusion was facilitated using a perfusion pump at 530 ml/min. After about 60 min of perfusion, the different obtained perfusates were collected to determine the glucose concentration in the final solution using the glucose oxidase-peroxidase method. The length of the jejunum was measured, and the results obtained are expressed in mg/cm/h which corresponds to the amount of the absorbed glucose in mg per the length of the segment (cm) per the perfusion time (min).

### 2.11. Statistical Analysis

The analysis was performed with ANOVA and with Student's *t*-test followed by the Tukey test with post hoc multiple comparison threshold 5%. The fraction components with a percentage higher than 5% of the total fraction were subjected to hierarchical cluster analysis (HCA) and principal component analysis (PCA) using SPSS v22.0 software. In the case of HCA, the dendrogram (tree) was produced using Ward's method of hierarchical clustering with squared Euclidean distance between fractions.

## 3. Results

### 3.1. Extraction and Chemical Composition of *Nigella sativa* Obtained Fractions

**Figure 1:**
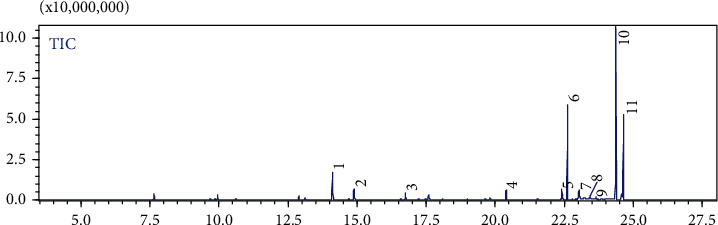
GC-MS total ion chromatogram (TIC) of the *NS* n-hexane fraction.

Table 1 gives the relative percentage of each component of the studied esterified n-hexane fraction according to their GC peak areas without correction factors. The different *NS* fractions were obtained using solvents of different polarities in a Soxhlet apparatus; the yields obtained were ranging from 2.53% to 21.51% (*w*/*w*). The GC-MS was used to investigate the chemical composition of the esterified fatty acid of the n-hexane fraction; the identification of the chemical compound present was assessed based on the comparison of their MS data with those stored on the National Institute of Standards and Technology (NIST147) computer library. The data represented in Table 1 and Figure 1 shows that the n-hexane fraction is with great richness of different compounds such as linoleic acid (44.65%), palmitic acid (16.32%), stearic acid (14.60%), thymoquinone (8.70%), and carvacrol (3.03%).

**Figure 2:**
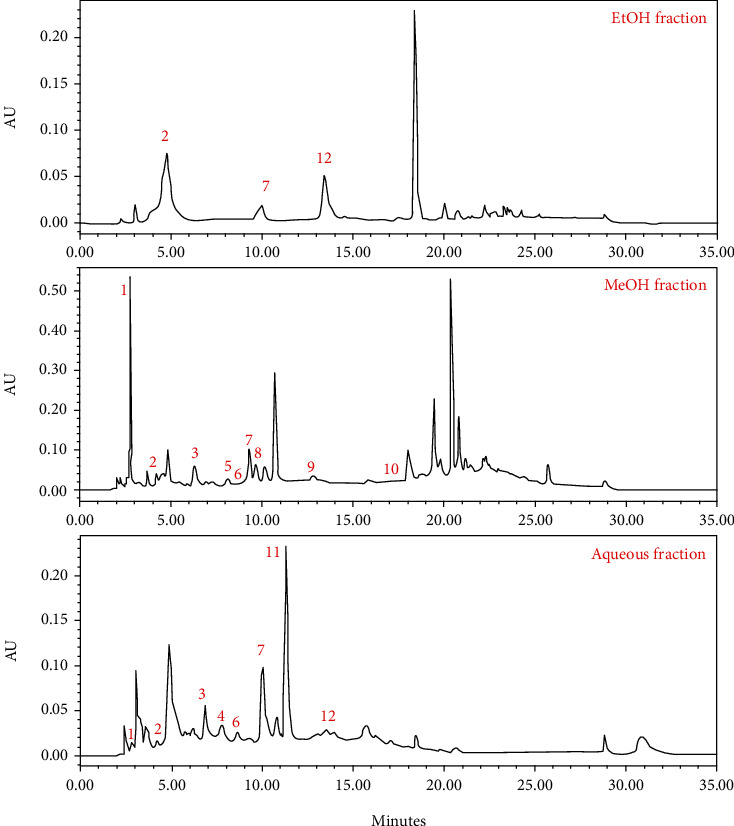
HPLC profile showing different peaks of *NS* fractions.

The chemical composition analysis of the EtOH, MeOH, and aqueous fractions was investigated using high-performance liquid chromatography; the results obtained are presented in Figure 2 and Tables 2(a) and 2(b). The EtOH fraction is rich mainly in three flavonoids: rutin, catechin, and kaempferol, while gallic acid, rutin, vanillic acid, and naringenin were among the molecules that we were able to identify in the MeOH fraction. Finally, the aqueous fraction was found to be rich in salicylic acid, rutin, ferulic acid, vanillin, and vanillic acid.

NF: not found, below the limit of detection. All values are represented by mean ± SD (*n* = 3).

### 3.2. Chemical Variation between the Different Fractions

The different molecules present in the different fractions and identified using HPLC were subject to hierarchical cluster analysis (HCA) and principal component analysis (PCA) in order to investigate the similarities existing between the different obtained fractions and relationship between the identified molecules and the pharmacological effect studied.

**Figure 3:**
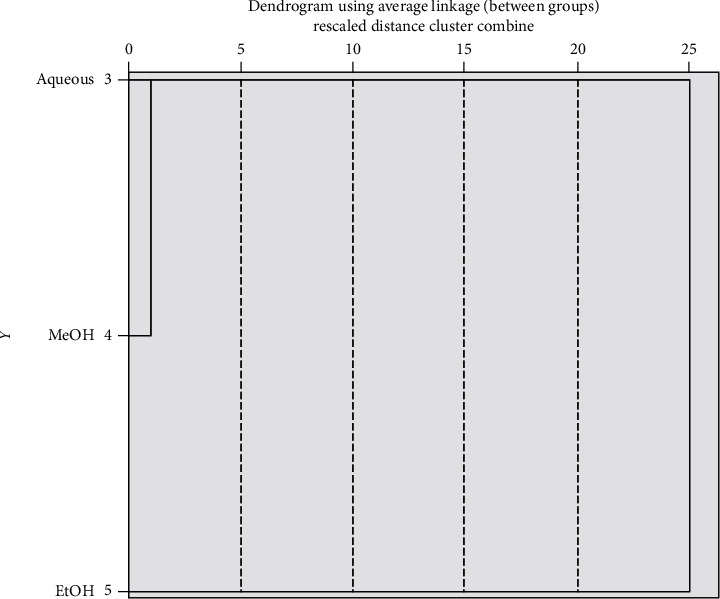
Dendrogram of three *NS* fractions produced by the hierarchical cluster analysis.

The results of the dendrogram represented in Figure 3 show that the NS fractions can be divided into two main clusters with a distance of 25 units. The fractions connected with short distance are more similar than those connected with large distance. The studied fractions can be divided into two main groups. The first group (Cluster 1) is represented by MeOH and aqueous fraction and characterized by the highest amount of gallic acid, catechin, rutin, and vanillic acid. The second group (Cluster 2) is represented by only the EtOH fraction that is rich with catechin, rutin, and kaempferol.

**Figure 4:**
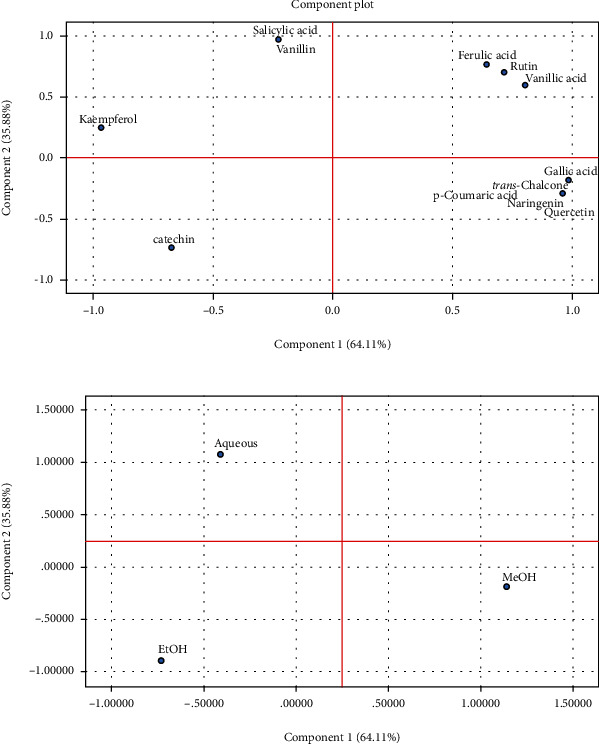
2D graphical representation of principal component analysis of chemical compositions of *NS* fraction. (a, b) PCA distributions of variables and samples, respectively.

As shown on Figure 4, the first principal component PC1 accounted for 64.11% of the total variance that correlates positively with gallic acid, vanillic acid, and rutin which were predominant components of our fractions. PC1 was found to correlate negatively with kaempferol, catechin, and salicylic acid. The second principal components accounted for 35.88% of the total variance, which positively correlated with salicylic acid. These PCA results show that the NS fractions can be classified into two main groups which confirm the finding represented in the HCA.

### 3.3. Acute Toxicity

The acute oral toxicity of the studied fractions represented in Table 3 has shown a nontoxic effect even at the highest dose administered (5 g/kg). Also, we mention that the *albino mice* have normal behavior after their force feeding. Therefore, there has been no harmful effect; for that, we can conclude that the different *NS* fractions obtained using the Soxhlet apparatus are nontoxic. The statistical analysis showed no significant difference between the control group and the tested fractions (*p* > 0.05).

### 3.4. In Vitro Study of *α*-Amylase

The *in vitro* inhibition ability of *α*-amylase of the different *NS* fractions was evaluated. It was observed that the different extracts were endowed with a capacity to inhibit *α*-amylase and were very effective at all doses tested (0.45, 0.9, and 1.82 mg/ml). It was also noted that the MeOH (0.103 mg/ml ± 0.015), EtOH (0.255 mg/ml ± 0.060), and aqueous (0.310 mg/ml ± 0.015) fractions have registered an IC_50_ value that was lower than that obtained by the acarbose (0.350 mg/ml ± 0.021) used as a control in the study (Table 4), while the dichloromethane and n-hexane fractions gave, respectively, IC_50_ values of 1.330 mg/ml ± 0.092 and 0.760 mg/ml ± 0.007 that were higher than the control.

**Figure 5:**
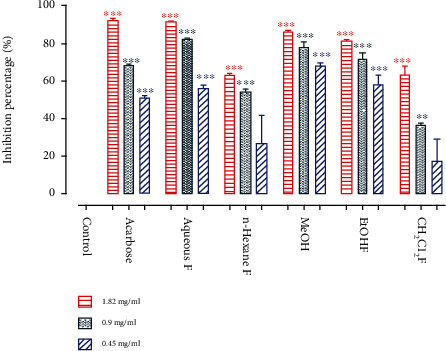
Inhibitory activity of *NS* fractions and acarbose (positive control) against *α*-amylase at different doses.

It was observed at 1.82 mg/ml has the highest inhibition percentage of 91.49% that was exhibited by the aqueous fraction, which was almost the same percentage obtained by the drug used (92.24%). The MeOH fraction had an inhibition activity of 86.39%, while the EtOH fraction showed an inhibition capacity of 81.28%. It was also noticed that the dichloromethane and n-hexane fractions gave approximately the same inhibition percentage 63.33% (Figure 5).

### 3.5. In Vivo *α*-Amylase Inhibition

**Figure 6:**
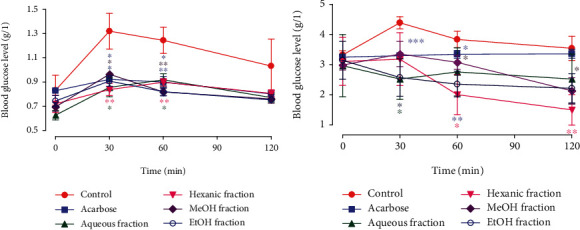
Effect of *NS* fractions on serum glucose level after starch loading in (a) normal and (b) diabetic rats.

Regarding the results of the *in vivo α*-amylase inhibition of normal and diabetic rats, they are depicted in Figures 6(a) and 6(b). In normal rats, it was observed that glycemia increases from 0.83 to 1.32 g/l after oral administration of starch in the control group. A significant decrease was observed compared to the control after the oral administration of the different fractions at a dose of 250 mg/kg at 30 and 60 min. At 120 min, no significant difference was observed between glycemia of the control group and the other groups, while in diabetic rats the glycemia of the control group increased from 3.16 to 4.35 g/l in alloxan diabetic rats after an oral administration of starch. The postprandial blood glucose decreases significantly in aqueous (*p* < 0.05) and EtOH (*p* < 0.05) fractions at a dose of 250 mg/kg, after 30 minutes of oral administration of starch, while after 60 min of treatment the glycemia decreased significantly in n-hexane (*p* < 0.05), MeOH (*p* < 0.05), and EtOH (*p* < 0.01) fractions and this decrease continues until 120 min of starch overload for MeOH (*p* < 0.05) and n-hexane fractions (*p* < 0.01).

### 3.6. In Situ Intestinal Glucose Absorption

**Figure 7:**
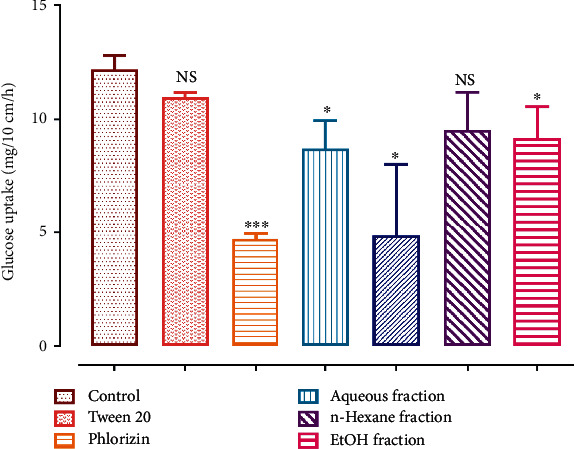
*In situ* intestinal glucose absorption of *NS* fractions.

The results obtained from the *in situ* study are described in Figure 7, where we observe that in the absence of the obtained fractions the intestinal glucose absorption reached 12.18 mg/10 cm/h. The intestinal glucose absorbed in the presence of the MeOH fraction was in the order of 4.85 mg/10 cm/h, followed by the aqueous fraction (8.70 mg/10 cm/h) and the EtOH fraction (9.15 mg/10 cm/h). The n-hexane fraction was not statistically significant to the phlorizin used as the positive control in our experiment. The phlorizin showed an important inhibiting effect on glucose intestinally absorbed which was equal to 4.71 mg/10 cm/h. Tween 20 (1%) which was used as a negative control showed no significant effect when compared to the control. The percentage of inhibition of the aqueous, MeOH, and EtOH fractions plus the phlorizin used as a control was, respectively, 28.54, 60.12, 24.82, and 61.29%.

### 3.7. Statistical Correlation

The statistical correlation depicted on Table 5 shows that IC_50_ correlates negatively with five chemical components (gallic acid, p-coumaric acid, naringenin, quercetin, kaempferol, and *trans*-chalcone). These different compounds act significantly on the IC_50_ value. The kaempferol was found to correlate negatively with the IC_50_ value.

Concerning the inhibition percentage, a high significant positive correlation with gallic acid principally and with other three compounds (p-coumaric acid, naringenin, quercetin, kaempferol, and *trans*-chalcone) was seen.

## 4. Discussion

The present study is aimed at investigating the inhibitory effect of the different obtained fractions from the Soxhlet apparatus on pancreatic *α*-amylase and intestinal glucose absorption. The best IC_50_ values obtained in the *in vitro* inhibition activity were chosen to be the subject of the *in vivo* inhibition of *α*-amylase activity on normal and alloxan diabetic rats. After that, the *in situ* intestinal glucose absorption pathway in normal rats was assessed. The results of this work prove that the different fractions obtained exhibited an *in vitro* inhibiting potential on the pancreatic enzyme *α*-amylase especially the aqueous, MeOH, EtOH, and n-hexane fractions that gave an IC_50_ value that in some fractions was lower than that obtained by the acarbose used as a control. Compared to our findings, it was reported in Value et al.'s [25] study that the aqueous extract was able to inhibit the *α*-amylase in a dose-dependent manner, and an inhibition capacity of about 84% at a dose of 100 mg/ml was observed, although, in our work, we found that it inhibits the enzyme with a percentage of 91.49% at a dose of 1.82 mg/ml. Our EtOH fractions gave an IC_50_ value of about 250 *μ*g/ml that was higher than that obtained in Sandhya and Kannayiram's [26] study, and it was equal to 100 *μ*g/ml. In Buchholz and Melzig's study [27], an inhibition percentage of 45% at a concentration of 2.5 mg/ml was registered which was less effective than that obtained in our results where the IC_50_ value was 100 *μ*g/ml. On the other side, the n-hexane fraction was found to inhibit *α*-amylase with an IC_50_ of 450 *μ*g/ml [28] which was lower than the IC_50_ found in our results (760 *μ*g/ml).

The *in vivo* study indicates that after a short time of starch administration, the different fractions were able to decrease the blood sugar level in normal and diabetic rats, which confirms the *in vitro* results. Varghese and Mehrotra [29] have reported that hydro-acetone extract of *NS* possesses an ability to inhibit *α*-amylase with an IC_50_ equal to 314.4 *μ*g/ml; this effect was potentialized when the extract was microencapsulated and the IC_50_ become lower 224.1 *μ*g/ml which enriches the *α*-amylase inhibitory activity. Moreover, it was shown that the methanolic and ethanolic extracts of *NS* have a lowering potential of the blood glucose and on lipid profile in alloxan diabetic rats while no regeneration of *β* cell islet was observed [30]. Another study by Khanam and Dewan [31] indicates that the aqueous and n-hexane extracts have normalized the serum glucose and lipid profile; also, it was shown that these extracts have a regenerating potential on the *β* cell pancreatic islet.

Concerning the *in situ* test, it was observed that the tested fractions and phlorizin, a specific inhibitor of SGLT1 and SGLT2, have shown a reduction of intestinal glucose absorption. SGLT1 is a glucose transporter found in the apical membrane of the small intestine and is responsible for glucose transportation from the intestinal lumen, while SGLT2 plays a crucial role in the glucose reabsorption in the proximal tubules [32]. The obtained results could be due to the presence of bioactive compounds that are endowed with inhibitory action on glucose transporters situated in the mucosa. In Meddah et al.'s study, it was showed that the aqueous extract of *NS* seeds using the short-circuit current technique possesses an inhibition activity of sodium-dependent d-glucose absorption that was dose dependent and at very lower doses (IC_50_ = 10 pg/ml) [33]. It was also indicated in the same study that the aqueous extract can control SGLT1 from the luminal side, which confirms the plant's ability to inhibit intestinal glucose absorption. Several studies have demonstrated that flavonoids such as catechins have the ability to inhibit the SGLT1 transporter in a competitive or noncompetitive way [34]. Tea polyphenols were indicated to be able to inhibit the SGLT1 activity which induced a reduction of the intestinal glucose absorbed [35].

The GC-MS results of the esterified fatty acid of n-hexane fraction and HPLC-UV analysis of the organic fractions revealed a great richness by different bioactive compounds such as thymoquinone, palmitic acid, ferulic acid, and rutin. Furthermore, in our previous study on *NS*, we demonstrated that the different fractions were rich with different bioactive compounds such as polyphenols and flavonoids [14]. Abdelmeguid et al. [36] in a study realized on streptozotocin-diabetic rats treated intraperitoneally with the aqueous extract, oil, and thymoquinone decrease diabetes with an elevation in serum insulin. It was also mentioned that the thymoquinone present in our n-hexane fraction by 8.70% and the aqueous fraction have played a major role in the amelioration of the different toxic effects starting from DNA damage to mitochondrial fragmentation and vacuolization induced by STZ, which indicate that thymoquinone is endowed with a protective effect against STZ by inducing a decrease of oxidative stress. In the results reported by [37], they found that *NS* oil showed its capacity to lower blood glucose in diabetic rats which suggests the antidiabetic potential of the *NS* oil.

The high-performance liquid chromatography analysis indicated the presence of several molecules of polyphenol and flavonoid nature that are known to have a great capacity to chelate the digestive enzymes such as *α*-amylase and a-glucosidase [38]. For example, ferulic acid, a phenolic compound present in our aqueous and MeOH fraction, respectively, by an amount of 0.042 ± 0.001 and 0.037 ± 0.0002 (mg/100 g of DW), has been mentioned to possess the ability to restore the parameters such as blood glucose, the insulin level in diabetic rats to normal by not only inhibiting gluconeogenesis and negative regulators of insulin but also by improving the hepatic glycogenesis [39]. It was also demonstrated by [40] that ferulic acid is an inhibitor of porcine *α*-amylase (IC_50_ = 0.622 mg/ml) by interacting with amino acid residues. Also, rutin, a flavonoid present in our aqueous and methanolic fractions with an amount of 21.46 ± 0.26 and 21.07 ± 0.10 mg/100 g, is found to be effective and able to stop the *α*-amylase activity with about 53% [41]. The naringenin found with 0.21 ± 0.001 mg/100 g of DW in MeOH fraction is also capable of inhibiting the pancreatic *α*-amylase [42].

Gallic acid, a phenolic compound present in our aqueous and MeOH fraction (2.39 ± 0.01 and 19.92 ± 0.015 mg/100 g), was found to be able to inhibit *α*-amylase with an IC_50_ = 1.09 *μ*g/ml [43]. Also, it was mentioned that its combination with acarbose showed a great synergic capacity to inhibit *α*-amylase and glucosidase [44]. It was recorded that kaempferol, a flavonoid present in the ethanolic fraction by 0.032 mg/100 g of DW, showed a higher inhibition capacity of *α*-amylase and this could be due to their high binding to the pancreatic enzyme [45]. Also, catechin, a molecule present with an important quantity in EtOH (89.03) followed by MeOH fraction (19.92), showed its capacity to inhibit the pancreatic *α*-amylase [46].

Furthermore, it was registered that two fatty acids present in n-hexane fraction, palmitic acid and linoleic acid with percentages, respectively, 44.65% and 16.32%, are found to be endowed with a weak to moderated ability to inhibit the pancreatic *α*-amylase [47]. In Aazza et al.'s study, they have suggested that carvacrol could play a crucial role in *α*-amylase inhibition [48]. Finally, it was also registered that the carvacrol present in *Zataria multiflora* has an inhibition capacity of 71% on pancreatic *α*-amylase [49].

## 5. Conclusion

The finding of the present study revealed that different *NS* fractions are endowed with a significant inhibitory effect on both *α*-amylase digestive enzyme and intestinal glucose absorption. This effect could be attributed to the different bioactive compounds identified using HPLC/UV and GC/MS such as gallic acid, p-coumaric acid, naringenin, quercetin, kaempferol, *trans*-chalcone, linoleic acid, and thymoquinone. More studies need to be done on these different fractions to confirm their antidiabetic activity and for their further use as an alternative of commercialized drugs.

## Figures and Tables

**Table 1:** Chemical constituents identified in the *NS* n-hexane fraction using GC-MS.

No.	Molecule name	Retention time	%
1	Thymoquinone	14.108	8.701
2	Carvacrol	14.883	3.038
3	Isocaryophillene	16.758	1.771
4	Myristic acid	20.400	3.112
5	Palmitoleic acid	22.417	2.945
6	Palmitic acid	22.617	16.321
7	Arachic acid	23.033	3.219
8	7-Hexadecenoic acid	23.442	0.954
9	Octadecanoic acid, 17-methyl	23.650	0.675
10	Linoleic acid	24.383	44.656
11	Stearic acid	24.650	14.608

**Table:** (a) Chemical components of *NS* fractions identified using HPLC

Peak no.	Polyphenolic compounds	Retention time	% area EtOH	% area MeOH	% area aqueous
1	Gallic acid	2.420	—	4.27	1.60
2	Catechin	4.178	31.38	0.92	1.26
3	Vanillic acid	6.837	—	2.34	5.63
4	Vanillin	7.757	—	—	3.71
5	p-Coumaric acid	8.132	—	0.76	—
6	Ferulic acid	8.604	—	0.71	2.18
7	Rutin	10.003	4.81	2.83	7.68
8	Naringenin	9.659	—	1.86	—
9	Quercetin	12.474	—	0.32	—
10	*trans-*Chalcone	17.771	—	0.47	—
11	Salicylic acid	11.308	—	—	16.44
12	Kaempferol	13.476	3.57	—	1.68

**Table:** (b) The polyphenolic compound content in *NS* seeds (mg/100 g of plant DW)

Peak no.	Polyphenolic compounds	EtOH fraction	MeOH fraction	Aqueous fraction
1	Gallic acid	NF	19.92 ± 0.015	2.39 ± 0.016
2	Catechin	89.03 ± 0.011	13.79 ± 0.053	6.87 ± 0.099
3	Vanillic acid	NF	4.36 ± 0.029	3.81 ± 0.022
4	Vanillin	NF	NF	0.007 ± 0.0004
5	p-Coumaric acid	NF	0.008 ± 3.78 *e*^−5^	NF
6	Ferulic acid	NF	0.037 ± 0.00025	0.0428 ± 0.001
7	Rutin	6.46 ± 0.004	21.07 ± 0.105	21.46 ± 0.26
8	Naringenin	NF	0.213 ± 0.001	NF
9	Quercetin	NF	0.00038 ± 4.43 *e*^−5^	NF
10	*trans*-Chalcone	NF	0.009 ± 9.1 *e*^−5^	NF
11	Salicylic acid	NF	NF	32.26 ± 0.094
12	Kaempferol	0.032 ± 4.75 *e*^−5^	NF	0.031 ± 0.0002

**Table 3:** Effects of different NS fractions on the bodyweight of *albino mice* with doses of 1, 2, and 5 g/kg. Data are expressed as mean ± SD (*n* = 6).

Fractions tested	Doses administered per os g/kg	Bodyweight		
		Initial body weight at the first day	Final body weight at day 14	Difference of weight
Aqueous F.	1	29.6 ± 0.75	28.05 ± 0.92	-1.55
	2	33.17 ± 2.46	32 ± 2.32	-1.17
	5	29.04 ± 1.23	28.32 ± 1.54	-0.72

MeOH F.	1	31.62 ± 1.08	30.82 ± 1.23	-0.8
	2	31.4 ± 0.97	31.32 ± 0.96	-0.08
	5	34.66 ± 1.19	34.04 ± 1.39	-0.62

EtOH F.	1	30.72 ± 0.66	29.75 ± 1.05	-0.97
	2	29.85 ± 1.12	30.25 ± 0.77	-0.4
	5	31 ± 1.5	30.8 ± 1	-0.2

n-Hexane F.	1	31.87 ± 0.81	30.55 ± 0.91	-1.32
	2	31.75 ± 1.02	31.15 ± 1.27	-0.6
	5	31.60 ± 0.54	30.64 ± 0.68	-0.96

Control		30.85 ± 1.02	29.62 ± 1.18	-1.23

**Table 4:** Different IC_50_ values of *NS* fractions and acarbose on pancreatic *α*-amylase.

Tested inhibitors	IC_50_ values (mg/ml)
Aqueous F.	0.31 ± 0.01
MeOH F.	0.10 ± 0.06
EtOH F.	0.25 ± 0.01
CH_2_CL_2_ F.	1.33 ± 0.09
n-Hexane F.	0.76 ± 0.01
Acarbose	0.35 ± 0.02

The fractions with the lowest IC_50_ value were chosen for the *in vivo α*-amylase test and *in situ* intestinal glucose absorption.

**Table 5:** Correlation coefficient between the chemical components of NS fractions and IC_50_ and inhibition%.

	Gallic acid	Catechin	Vanillin	p-Coumaric acid	Ferulic acid	Rutin	Naringenin	Salicylic acid	Quercetin	Kaempferol	*trans-*Chalcone	Vanillic acid
IC_50_	-0.841^∗∗^	0.169	0.634	-0.872^∗∗^	-0.130	-0.218	-0.872^∗∗^	0.636	-0.871^∗∗^	0.861^∗∗^	-0.873^∗∗^	-0.335
Inhibition %	1.000^∗∗^	-0.517	-0.414	0.995^∗∗^	0.478	0.562	0.995^∗∗^	-0.415	0.989^∗∗^	-0.998^∗∗^	0.995^∗∗^	0.670^∗^

## Data Availability

All data used to support the finding of our study are available from the corresponding author upon request.

## References

[1] Raffel L. J., Goodarzi M. O. (2013). *Diabetes Mellitus*.

[2] Raffel L. J., Theatres P., Angeles L. (2014). *Diabetes Mellitus*.

[3] Bahmani M., Golshahi H., Saki K., Rafieian-Kopaei M., Delfan B., Mohammadi T. (2014). Medicinal plants and secondary metabolites for diabetes mellitus control. *Asian Pacific Journal of Tropical Disease*.

[4] Smith M. E., Morton D. G. (2010). Digestion and absorption. *The Digestive System*.

[5] Oyedemi S. O., Oyedemi B. O., Ijeh I. I., Ohanyerem P. E., Coopoosamy R. M., Aiyegoro O. A. (2017). Alpha-amylase inhibition and antioxidative capacity of some antidiabetic plants used by the traditional healers in southeastern Nigeria. *Scientific World Journal*.

[6] Patel M., Mishra S. (2012). A kinetic study for in-vitro intestinal uptake of monosaccharide across rat everted gut sacs in the presence of some antidiabetic medicinal plants. *The Internet Journal of Alternative Medicine*.

[7] Shabana A., El-Menyar A., Asim M., Al-Azzeh H., Al Thani H. (2013). Cardiovascular benefits of black cumin (Nigella sativa). *Cardiovascular Toxicology*.

[8] Majdalawieh A. F., Fayyad M. W. (2015). Immunomodulatory and anti-inflammatory action of Nigella sativa and thymoquinone: a comprehensive review. *International Immunopharmacology*.

[9] Khan M. A., Chen H. C., Tania M., Zhang D. Z. (2011). Anticancer activities of Nigella sativa (black cumin). *African Journal of Traditional, Complementary and Alternative Medicines*.

[10] Majdalawieh A. F., Fayyad M. W. (2016). Recent advances on the anti-cancer properties of Nigella sativa, a widely used food additive. *J. Ayurveda Integr. Med.*.

[11] Mahmoudvand S. A., Sepahvand H., Jahanbakhsh A., Ezatpour S., Ayatollahi Mousavi B. (2014). Évaluation de l'activite antifongique de l'huile essentielle et de divers extraits de Nigella sativa et de son composant principal, la thymoquinone, contre des dermatophytes pathog enes. *Journal de Mycologie Médicale*.

[12] Keyhanmanesh R., Gholamnezhad Z., Boskabady M. H. (2014). The relaxant effect of Nigella sativa on smooth muscles, its possible mechanisms and clinical applications. *Iranian Journal of Basic Medical Sciences*.

[13] Kadam D., Lele S. S. (2017). Extraction, characterization and bioactive properties of Nigella sativa seedcake. *Journal of Food Science and Technology*.

[14] Dalli M., Azizi S., Kandsi F., Gseyra N. (2021). Evaluation of the in vitro antioxidant activity of different extracts of Nigella sativa L. seeds, and the quantification of their bioactive compounds. *Mater. Today Proc.*.

[15] Deloer S., Bari M., Hoque M. M. (2019). Antibacterial properties of essential oil (EO) extracted from Nigella sativa Linn (black cumin) and its application against Vibrio cholerae in ground chicken meat. *Bangladesh Journal of Microbiology*.

[16] Dalli M., Azizi S. E., Benouda H. (2021). Molecular composition and antibacterial effect of five essential oils extracted from Nigella sativa L. seeds against multidrug-resistant bacteria: a comparative study. *Evidence-Based Complementary and Alternative Medicine*.

[17] Hassan S. T. S., Šudomová M. (2020). Comment on: Effects of Nigella Sativa on Type-2 Diabetes Mellitus: A Systematic Review. *International Journal of Environmental Research and Public Health*.

[18] El Rabey H. A., Al-Seeni M. N., Bakhashwain A. S. (2017). The antidiabetic activity of Nigella sativa and propolis on streptozotocin-induced diabetes and diabetic nephropathy in male rats. *Evidence-Based Complementary and Alternative Medicine*.

[19] Aïssi V. M., Soumanou M. M., Tchobo F. P., Kiki D. (2009). Etude comparative de la qualité des huiles végétales alimentaires raffinées en usage au Bénin. *Bull. d’Informations La Société Ouest Africaine Chim.*.

[20] National Institutes of Health (1985). *Guide for the Care and Use of Laboratory Animals*.

[21] Thalapaneni N. R., Chidambaram K. A., Ellappan T., Sabapathi M. L., Mandal S. C. (2008). Inhibition of Carbohydrate Digestive Enzymes by Talinum portulacifolium (Forssk) Leaf Extract. *Journal of Complementary and Integrative Medicine*.

[22] OECD (2008). Acute Oral Toxicity-Up and Down Procedure (UDP). *OECD Guidelines for the Testing of Chemicals*.

[23] Prince P. S., Menon V. P., Pari L. (1998). Hypoglycaemic activity of Syzigium cumini seeds: effect on lipid peroxidation in alloxan diabetic rats. *Journal of Ethnopharmacology*.

[24] Ponz F., Ilundain A., Lluch M. (1979). Method for successive absorptions with intestinal perfusion in vivo. *Revista Española de Fisiología*.

[25] Value S. J. R. I., Sathiavelu A., Sangeetha S., Archit R., Mythili S. (2013). In vitro anti-diabetic activity of aqueous extract of the medicinal plants Nigella sativa, Eugenia jambolana, Andrographis paniculata and Gymnema sylvestre. *International Journal of Drug Development & Research*.

[26] Sandhya A., Kannayiram G. (2020). Pharmacological, bioactive screening of medicinal plant Nigella sativa and the derived compound thymoquinone: an in vitro study. *International Journal of Research in Pharmaceutical Sciences*.

[27] Buchholz T., Melzig M. F. (2016). Medicinal plants traditionally used for treatment of obesity and diabetes mellitus-screening for pancreatic lipase and *α*-amylase inhibition. *Phytotherapy Research*.

[28] Bonesi M., Saab A. M., Tenuta M. C. (2020). Screening of traditional Lebanese medicinal plants as antioxidants and inhibitors of key enzymes linked to type 2 diabetes. *Plant Biosyst.*.

[29] Varghese L. N., Mehrotra N. (2020). *α*-Amylase inhibitory activity of microencapsulated Nigella sativa L and herb-drug interaction: an in vitro analysis. *Annals of Phytomedicine: An International Journal*.

[30] Ikram F., Hussain F. (2014). Antidiabetic efficacy of Nigella sativa Linn in alloxan-induced diabetic rabbits. *IIUM Medical Journal Malaysia*.

[31] Khanam M., Dewan Z. F. (2009). Effects of the crude and the n-hexane extract of Nigella sativa Linn (kalajira) upon diabetic rats. *Bangladesh Journal of Pharmacology*.

[32] VENNESLAND B. (1948). Carbohydrate metabolism. *Annual Review of Biochemistry*.

[33] Meddah B., Ducroc R., El Abbes Faouzi M. (2009). Nigella sativa inhibits intestinal glucose absorption and improves glucose tolerance in rats. *Journal of Ethnopharmacology*.

[34] Chen C. H., Hsu H. J., Huang Y. J., Lin C. J. (2007). Interaction of flavonoids and intestinal facilitated glucose transporters. *Planta Medica*.

[35] Kobayashi Y., Suzuki M., Satsu H. (2000). Green tea polyphenols inhibit the sodium-dependent glucose transporter of intestinal epithelial cells by a competitive mechanism. *Journal of Agricultural and Food Chemistry*.

[36] Abdelmeguid N. E., Fakhoury R., Kamal S. M., Al Wafai R. J. (2010). Effects of Nigella sativa and thymoquinone on biochemical and subcellular changes in pancreatic *β*-cells of streptozotocin-induced diabetic rats. *Journal of Diabetes*.

[37] Akhtar M. T., Qadir R., Bukhari I. (2020). Antidiabetic potential of Nigella sativa L seed oil in alloxaninduced diabetic rabbits. *Tropical Journal of Pharmaceutical Research*.

[38] Wang Z., Gao X., Li W., Tan S., Zheng Q. (2020). Phenolic content, antioxidant capacity, and *α*-amylase and *α*-glucosidase inhibitory activities of Dimocarpus longan Lour. *Food Science and Biotechnology*.

[39] Narasimhan A., Chinnaiyan M. (2009). Ferulic acid exerts its antidiabetic effect by modulating insulin signalling molecules in the liver of high fat diet and fructose – induced type-2 diabetic adult male rat. *Applied Physiology, Nutrition, and Metabolism*.

[40] Zheng Y., Tian J., Yang W. (2020). Inhibition mechanism of ferulic acid against *α*-amylase and *α*-glucosidase. *Food Chemistry*.

[41] Dubey S., Ganeshpurkar A., Ganeshpurkar A., Bansal D., Dubey N. (2017). Glycolytic enzyme inhibitory and antiglycation potential of rutin. *Futur. J. Pharm. Sci.*.

[42] Tadera K., Minami Y., Takamatsu K., Matsuoka T. (2006). Inhibition of .alpha.-glucosidase and .alpha.-amylase by flavonoids. *Journal of Nutritional Science and Vitaminology (Tokyo)*.

[43] Adefegha S. A., Oboh G., Ejakpovi I. I., Oyeleye S. I. (2015). Antioxidant and antidiabetic effects of gallic and protocatechuic acids: a structure–function perspective. *Comparative Clinical Pathology*.

[44] Oboh G., Ogunsuyi O. B., Ogunbadejo M. D., Adefegha S. A. (2016). Influence of gallic acid on *α*-amylase and *α*-glucosidase inhibitory properties of acarbose. *Journal of Food and Drug Analysis*.

[45] Liao L., Chen J., Liu L., Xiao A. (2018). Screening and binding analysis of flavonoids with alpha-amylase inhibitory activity from lotus leaf. *Journal of the Brazilian Chemical Society*.

[46] Matsumoto N., Ishigaki F., Ishigaki A., Iwashina H., Hara Y. (1993). Reduction of blood glucose levels by tea catechin. *Bioscience, Biotechnology, and Biochemistry*.

[47] Su C. H., Lai M. N., Ng L. T. (2013). Inhibitory effects of medicinal mushrooms on *α*-amylase and *α*-glucosidase – enzymes related to hyperglycemia. *Food & Function*.

[48] Aazza S., El-Guendouz S., Miguel M. G. (2016). Antioxidant, anti-inflammatory and anti-hyperglycaemic activities of essential oils from thymbra capitata, thymus albicans, thymus caespititius, thymus carnosus, thymus lotocephalus and thymus mastichina from Portugal. *Natural Product Communications*.

[49] Kamrani Y. Y., Amanlou M., Yazdanyar A., Adli Moghaddam A., Ebrahimi S. N. (2008). Potential anti-diabetic and anti-oxidant activity of essential oil of Zataria multiflora leaves. *Planta Medica*.

